# Supplementary material for: A modelling study highlights the power of detecting and isolating asymptomatic or very mildly affected individuals for COVID-19 epidemic management
Source: BMC Public Health. 2020 Nov 27;20:1809. doi: 10.1186/s12889-020-09843-7 (PMC7691976; doi:10.1186/s12889-020-09843-7)
Supplement: Supplementary file 1 — Additional file 1. [file 12889_2020_9843_MOESM1_ESM.pdf]

Supplementary Information for A modelling study highlights the power of detecting and isolating asymptomatic or very mildly affected individuals for COVID-19 epidemic management.

Lía Mayorga, Clara García Samartino, Gabriel Flores, Sofía Masuelli, María V. Sánchez, Luis S. Mayorga and Cristián G. Sánchez

1. Computation of the effective reproduction number R_e

The basic reproduction number for an arbitrary compartmental model can be obtained following the method described in [1]. Given the system of differential equations

$$
\begin{aligned}
\dot{H} &= -\beta I_S S - \beta' I_A S \\
\dot{E} &= \beta I_S S + \beta' I_A S - \mu E \\
\dot{I}_A &= p_A \mu E - (1 - p_{AI})(1/D_{recA}) I_A - p_{AI}(1/D_A) I_A \\
\dot{I}_S &= (1 - p_A)\mu E - \gamma I_S \\
\dot{A} &= p_{AI}(1/D_A) I_A - (1/(D_{recA} - D_A)) A \\
\dot{L} &= (1 - p_H)\gamma I_S - (1/D_{rec}) L \\
\dot{O} &= p_H \gamma I_S - (1 - p_{ICU})(1/D_{rec}) O - p_{ICU}(1/D_{lag}) O \\
\dot{H}_{ICU} &= p_{ICU}(1/D_{lag}) O - (1 - p_F)(1/D_{recICU}) H_{ICU} - p_F(1/D_{recICU}) H_{ICU} \\
\dot{R}_A &= (1 - p_{AI})(1/D_{recA}) I_A + (1/(D_{recA} - D_A)) A \\
\dot{R}_S &= (1/D_{rec}) L + (1 - p_{ICU})(1/D_{rec}) O + (1 - p_F)(1/D_{recICU}) H_{ICU} \\
\dot{F} &= p_F(1/D_{recICU}) H_{ICU}
\end{aligned}
$$

with $\beta = R_0(1/D_{inf})$, $\beta' = f_A R_0(1/D_{inf})$, $\mu = (1/D_{inc})$ y $\gamma = (1/D_{inf})$ the effective reproduction number R_e can be calculated as follows:

Assuming with no loss of generality a disease free state corresponding to $S = 1$ and all other variables equal to zero (x_0) we have that, for the variables

E, I_A and I_S, in the standard notation

$$\mathcal{F}(x_0) = \begin{pmatrix} \beta I_S + \beta' I_A \\ 0 \\ 0 \end{pmatrix}$$

and

$$\mathcal{V}(x_0) = \begin{pmatrix} -\mu E \\ p_A \mu E - ((1 - p_{AI}/D_{rec} - p_{AI}/D_A) I_A \\ (1 - p_A)\mu E - \gamma I_S \end{pmatrix}$$

The corresponding Jacobian matrices are:

$$F = \begin{bmatrix} 0 & \frac{R_0 f_A}{D_{inf}} & \frac{R_0}{D_{inf}} \\ 0 & 0 & 0 \\ 0 & 0 & 0 \end{bmatrix}$$

and

$$V = \begin{bmatrix} -\mu & 0 & 0 \\ p_A \mu & -((1 - p_{AI}/D_{rec} - p_{AI}/D_A) & 0 \\ (1 - p_A)\mu & 0 & -\gamma \end{bmatrix}$$

The effective reproduction number is calculated as the largest eigenvalue of the next generation matrix FV^{-1} obtaining

$$R_e = R_0 \left(1 - p_A\right) + \frac{R_0 f_A p_A}{D_{inf} \left(\frac{1 - p_{AI}}{D_{recA}} + \frac{p_{AI}}{D_A} \right)}.$$

Note that for the case in which $p_A = 0$ $R_e = R_0$ as expected. With no isolation of asymptomatic patients, that is with $p_{AI} = 0$, R_e might be larger than R_0 since asymptomatic individuals remain infective for a time that is larger than D_{inf}.

Referencias

[1] Van den Driessche, P., and Watmough, J. (2002). Reproduction numbers and sub-threshold endemic equilibria for compartmental models of disease transmission. Mathematical Biosciences, 180(1-2), 29-48. http://doi.org/10.1016/S0025-5564(02)00108-6
